# Pattern recognition receptors as key players in adrenal gland dysfunction during sepsis

**DOI:** 10.1186/cc11734

**Published:** 2012-11-14

**Authors:** THN Tran, W Kanczkowski, V Hoesker, SR Bornstein, K Zacharowski

**Affiliations:** 1 Hospital of the Goethe-University Frankfurt, Clinic of Anaesthesiology Intensive Care Medicine and Pain Therapy, Frankfurt am Main, Germany; 2 Technical University Dresden, Germany

## Background

Undergoing systemic inflammation, the innate immune system releases excessive proinflammatory mediators, which finally can lead to organ failure. Pattern recognition receptors (PRRs), such as Toll-like receptors (TLRs) and NOD-like receptors (NLRs), form the interface between bacterial and viral toxins and innate immunity. During sepsis, patients with diagnosed adrenal gland insufficiency are at high risk of developing a multiorgan dysfunction syndrome, which dramatically increases the risk of mortality. To date, little is known about the mechanisms leading to adrenal dysfunction under septic conditions. Here, we investigated the sepsis-related activation of the PRRs, cell inflammation, and apoptosis within adrenal glands.

## Methods

Two sepsis models were performed: the *polymicrobial sepsis model (caecal ligation *and *puncture (CLP)) and the *LTA-induced intoxication model. All experiments received institutional approval by the Regierungspräsidium Darmstadt. CLP was performed as previously described [1], wherein one-third of the caecum was ligated and punctured with a 20-gauge needle. For LTA-induced systemic inflammation, TLR2 knockout (TLR2^-/-^) and WT mice were injected intraperitoneally with pure LTA (pLTA; 1 mg/kg) or PBS for 2 hours. To detect potential direct adrenal dysfunction, mice were additionally injected with adrenocorticotropic hormone (ACTH; 100 μg/kg) 1 hour after pLTA or PBS. Adrenals and plasma samples were taken. Gene expressions in the adrenals (rt-PCR), cytokine release (multiplex assay), and the apoptosis rate (TUNEL assay) within the adrenals were determined.

## Results

**Figure 1:**
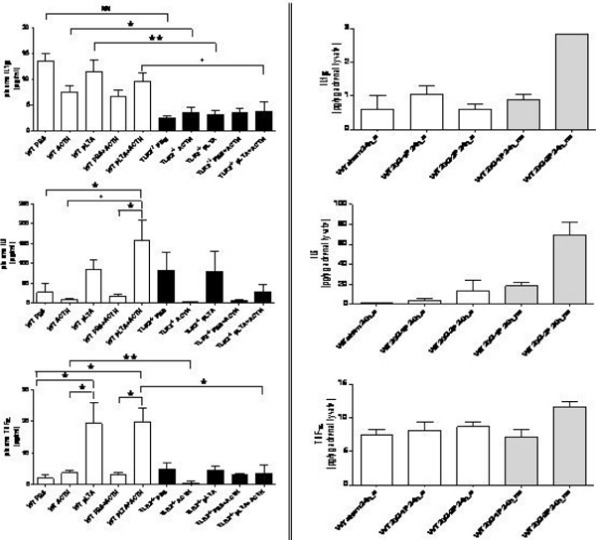
**Cytokine concentrations of IL-1β, IL-6 and TNFα in plasma and adrenal lysates**. From WT and TLR2^-/- ^mice treated with PBS (2 hours), ACTH (100 μg/kg; 1 hour), pLTA (1 mg/kg; 2 hours) or PBS/ACTH and pLTA/ACTH (left) and from WT mice that underwent CLP sham operation (sham), CLP with single (CLP-1P) or double (CLP-2P) puncture (right). Animals that survived after 24-hour CLP are labelled 's' and deceased ones 'ns'. Data are presented as mean ± SEM (*n *≥ 6). Statistical significance was determined by one-way ANOVA and Bonferroni's post test (vs. PBS within one group) or *t *test (WT vs. TLR2^-/-^). *P < 0.05, **P < 0.01, +*P *< 0.005, ++*P *< 0.001, #*P *< 0.0005, ##*P *< 0.0001.

**Figure 2:**
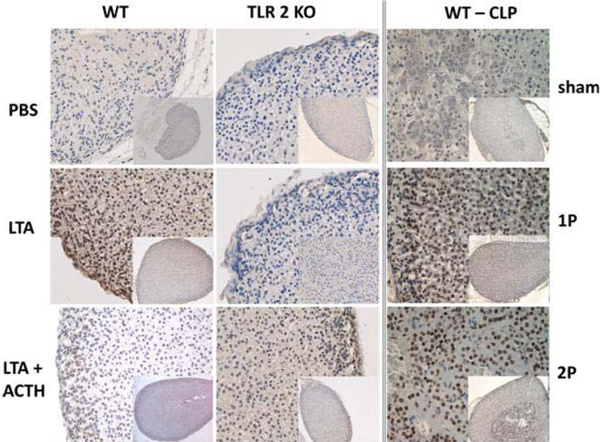
**Apoptosis in murine adrenal glands**. From WT and TLR2^-/- ^mice treated with PBS (2 hours), pLTA (1 mg/kg; 2 hours) or pLTA/ACTCH (left) and from WT mice that underwent CLP sham operation (sham), CLP with single (CLP-1P) or double (CLP-2P) puncture (right). TUNEL staining in adrenal sections showing whole adrenals (inserted pictures; 100× magnification), cortex and medulla area (400× magnification). The apoptotic cells appeared with brown nuclear staining.

In both models, adrenals showed increased mRNA expression of TLR2 and TLR4, various NLRs, cytokines as well as inflammasome components, NADPH oxidase subunits, and nitric oxide synthases (data not shown). In WT mice, ACTH alone had no effect on inflammation, while pLTA or pLTA/ACTH administration showed increased levels of the cytokines IL-1β, IL-6, and TNFα. TLR2^-/- ^mice indicated no response as expected (Figure 1, left). Interestingly, surviving CLP mice showed no inflammatory adrenal response, whereas nonsurvivors had elevated cytokine levels (Figure 1, right). Additionally, we identified a marked increase in apoptosis of both chromaffin and steroid-producing cells in adrenal glands obtained from mice with sepsis as compared with their controls (Figure 2).

## Conclusion

Taken together, sepsis-induced activation of the PRRs *may *contribute to adrenal impairment by enhancing tissue inflammation, oxidative stress and culminate in cellular apoptosis, *while mortality seems to be associated with adrenal inflammation*.
